# Management of Lipid Abnormalities in Patients with Diabetes

**DOI:** 10.1007/s11886-019-1246-1

**Published:** 2019-11-22

**Authors:** Anne Sillars, Naveed Sattar

**Affiliations:** The Institute of Cardiovascular and Medical Sciences, University of Glasgow, BHF Glasgow Cardiovascular Research Centre, 126 University Place, Glasgow, G12 8TA UK

**Keywords:** Dyslipidemia, Type 2 diabetes, Type 1 diabetes, Statins, Ezetimibe, PCSK9 inhibitors

## Abstract

**Purpose of Review:**

To describe lipid abnormalities in diabetes, when they occur and the evidence base for lipid management with established and new drugs to prevent diabetes complications. We also discuss how to manage statin intolerance.

**Recent Findings:**

Statins remain first-line therapy in patients with diabetes, though newer therapies to reduce LDL-C have emerged, including ezetimibe as an add-on therapy to statins, and injectable PCSK9 inhibitors, both of which are safe and effective in diabetes. Emerging evidence suggests a need to consider lipid-lowering therapies more often in younger patients with both type 1 and type 2 diabetes.

**Summary:**

Statins remain the cornerstone of lipid management in diabetes but other options are increasing. There is also now evidence for better managing apparent statin intolerance. Notably, younger patients lose the most life years from their diabetes, an observation that future guidelines need to consider.

## Introduction

Diabetes is a well-recognized risk factor for cardiovascular disease (CVD) and while hyperglycemia enhances risk, such patients are often obese, have hypertension, and are characterized by a specific dyslipidemia, such that the CVD risk in diabetes is multifactorial. Notably, one could say that nearly all patients with type 2 diabetes (T2DM) have lipid abnormalities. However, only in the late 1990s to 2000s, after major statin trials and relevant guideline changes, did we start to aggressively treat these lipid abnormalities, at least in high-income countries. For the next two decades or so, statins formed the cornerstone of lipid-lowering therapy in diabetes. This statin-dominated pattern has not changed, but additional ways to lessen LDL-C have emerged, and thus doctors have more therapies in their armory to treat dyslipidemia (Table 1). Furthermore, there is a push for some particularly high-risk patients to be treated more aggressively. There is also discussion surrounding when and how to treat younger patients with T2DM who have particularly elevated risk for CVD and greater premature mortality. Finally, although we focus mostly on lipids in T2DM, emerging data suggest we may be undertreating lipid levels in people with type 1 diabetes (T1DM), who lose on average more life years than those with T2DM. These aspects are discussed in more detail in this short review of the field.

**Table 1:** Summary of trial evidence for lipid lowering in type 2 diabetes

Classification of drug	Key trials	Findings	Clinical implications
Statins	CTT	LDL-C reduction of 1 mmol/L results in approximately 23% reduction in CV event. Intensive statin regimes result in statistically significant 15% further reduction in major vascular events, without significant side effects.	Statins as first line in patients with diabetes. Nowadays, the most commonly used are atorvastatin and rosuvastatin. Both have greater benefits on TG reduction than the older simvastatin and pravastatin.
Ezetimibe	IMPROVE-IT	Reduced CV mortality, major CV event and stroke by 5.5% absolute RR (hazard ratio, 0.85; 95% confidence interval, 0.78–0.94) The largest relative reductions occurred in patients with DM were in MI (24%) and stroke (39%).	First add-on therapy if patients are not reaching targets for LDL-c or non-HDL-c despite maximally tolerated statin therapy
PCSK9 inhibitor	FOURIER	Evolocumab reduced cardiovascular outcomes in patients with diabetes: HR 0.83 (95% CI 0.75–0.93; *p* = 0.0008) for primary composite endpoint. Similar data for Alirocumab.	Currently reserved for patients at very high absolute risk for CVD. This includes patients with FH or existing CVD, with sustained elevations in LDL-c despite maximally tolerated statin therapy plus ezetimibe.
Fibrates	ACCORD	Modest changes seen in the reduction of TG levels and increase in HDL-C levels.	Add-on to statins for mixed hyperlipidemia, without robust evidence demonstrating improved outcomes in CVD risk. Further ongoing trials with newer fibrates.
Icosapent ethyl; Eicosapentaenoic acid (EPA) ethyl ester	REDUCE-IT	Primary endpoint event occurred in 17.2% of treated patients compared with 22.0% in placebo group (HR 0.75; 95% CI 0.68 to 0.83; *p* < 0.001)	Potential new therapy with modest lowering of TG levels. Outcome benefits may be largely independent of TG lowering. Ongoing trials of similar agents should help reveal mechanisms in due course.
Bempedoic acid; ATP citrate lyase inhibitor	CLEAR-Harmony	Treatment reduced the mean LDL cholesterol level − 16.5% from baseline (difference vs. placebo in change from baseline, − 18.1 percentage points; 95% CI, − 20.0 to − 16.1; *p* < 0.001).	Potential new therapy for LDL cholesterol lowering

### What Are the Lipid Abnormalities in T2DM?

Dyslipidemia is an important biochemical abnormality in patients with T2DM, because of its independent association with an increased risk of morbidity and mortality from CVD [1–3]. Characteristically, these lipid abnormalities are high fasting and postprandial plasma triglyceride (TG) concentration, low levels of high density lipoprotein cholesterol (HDL-C), and normal or slightly increased concentration of low-density lipoprotein cholesterol (LDL-C), and increased numbers of small dense LDL (sdLDL) particles. Apolipoprotein B (ApoB), a carrier protein in both LDL and VLDL, is also raised. Increased mobilization of free fatty acids (FFA) from adipose tissue, in addition to impaired insulin-mediated skeletal muscle uptake, results in increased circulating FFA levels, increased hepatic fatty acid influx, and TG formation [4, 5].

Traditionally, LDL size and density are linked with CVD [6, 7], as well as LDL particle concentration, with small dense LDL particles postulated as having the greatest contribution to atherogenic disease. The etiology of this has shown to be multifactorial with in vitro studies demonstrating reductions in LDL receptor affinity [8], increased arterial wall binding of LDL [9], and altered LDL oxidation [10], each contributing to increased CVD risk. Reduced HDL concentration is usually characterized by reduced levels of subspecies HDL_2_ and is often associated with high TG levels [11]. Patients with T2DM are also likely to have smaller HDL particles.

However, another way to view lipid-associated risk in T2DM stems from genetic evidence from Ference et al. In their recent work, they compared the association of triglyceride-lowering LPL variants and LDL-C–lowering LDLR variants with the risk of CVD per unit difference in Apo B, to demonstrate that reducing both TG and LDL-C variants was associated with lower CHD risk per unit lower level of Apo B-containing lipoproteins [12]. Thus, it appears that the number of ApoB containing particles is the most important predictor of CVD risk, and therefore in patients with T2DM with raised TG levels, ApoB tends to be higher (due to accumulation of VLDL particles and smaller dense LDL) resulting in a raised lipid-associated risk for CVD. Whether apo B should be recommended for risk assessment in people with diabetes is a topical issue. It is our view that routine measurement of apo B is not presently needed given that a diagnosis of diabetes itself warrants lipid lowering and that non-HDL-cholesterol, which is high in diabetes, is available as a secondary lipid target. In recent analyses of UK biobank data, we showed that once total cholesterol and HDL-cholesterol were factored into a cardiovascular risk score, apo B adds little further predictive value [13]. We recognise some researchers remain strongly in favour of measuring apo B  buts its far greater cost and limited added prediction benefit, means its addition is unlikely to be cost effective.

### When Evidence Base for Statin Use in Diabetes Became Unquestionable

Throughout the 1990s and 2000s, a body of evidence demonstrating the benefits of statin therapy, both in the general population and in patients with diabetes, was created. The trials conducted had differing recruitment criteria, different outcomes with varying analyses and different drugs were studied. In 2005, the Cholesterol Treatment Trialists’ (CTT) Collaboration published a prospective meta-analysis utilizing data from 14 randomized trials of statin therapy, including data from 90,056 individuals [14]. With the increased power from the combined data, they showed that statin therapy statistically reduced LDL-C, and therefore CVD risk, over 5 years in an approximately linear fashion. Overall, LDL-C was approximately 0.8 mmol/L lower at 5 years in patients treated with statins. CTT demonstrated that a reduction of LDL-C of 1 mmol/L at 5 years result in an approximately 23% reduction in CV event, and that ultimately, the absolute benefit to be gained is based upon the individual’s baseline risk. Therefore, patients with T2DM stand to benefit more than those without diabetes from statin therapy. In 2010, the CTT collaboration furthered their analyses to assess the benefits of intensive LDL cholesterol lowering with statin therapy [15]. They looked at studies with an intensive versus standard regime (five studies) and those with a statin versus control regimes (21 studies). Intensive regimes resulted in a statistically significant 15% further reduction in major vascular events (*p* < 0.0001) compared with standard regimes, and the mean further reduction in LDL-C at 1 year was 0.51 mmol/L. Across all patients studied, further reductions in LDL-C resulted in the reduction of incident CV events by approximately one fifth for each 1.0 mmol/L reduction. These findings occurred without an increase in side effects, and the CTT collaboration has subsequently published a statement highlighting statin safety and tolerability [16].

For these reasons, statins are considered first-line treatments for many with T2DM, even at diagnosis, and in those patients with multiple risk factors for CVD, high-dose statin is recommended (Table 1).

### Other Proven Cardioprotective Cholesterol-Lowering Therapies in Diabetes

#### Ezetimibe

**Fig. 1:**
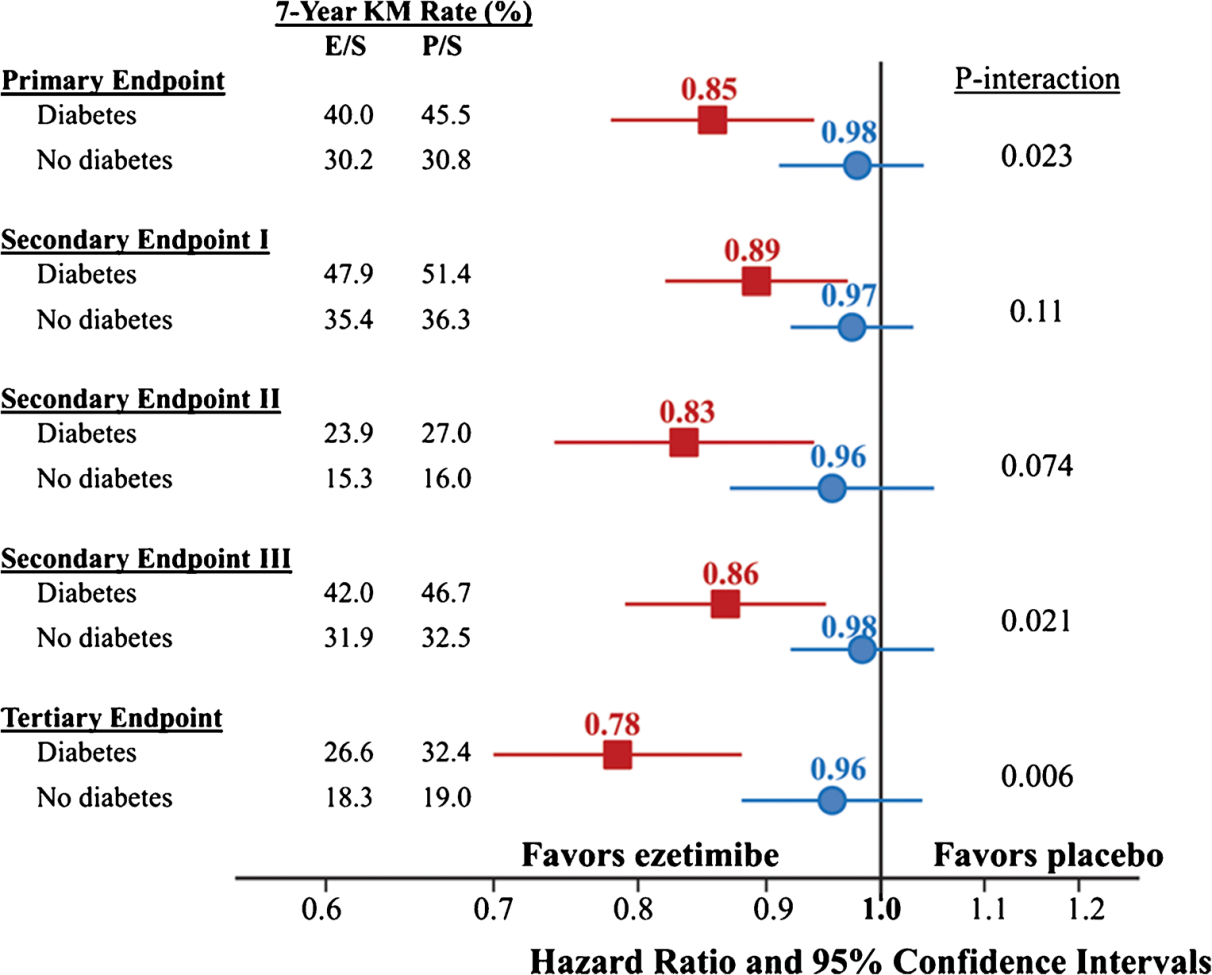
Ezetimibe composite efficacy outcomes by treatments and diabetes status*.* (With permission from: Giugliano RP, et al. Circulation 2018;137:1571–1582) [22••]

Ezetimibe selectively inhibits dietary and biliary cholesterol resulting in a reduction in LDL-C, a very modest increase in HDL-C (1–3%), and has no effect on TG. Prior to 2015, the evidence base for CV risk reduction with ezetimibe was equivocal [17–19], with the ENHANCE-trial failing to show improved CV outcomes [20]. However, in 2015, the Improved Reduction of Outcomes: Vytorin Efficacy International Trial (IMPROVE-IT) which randomized 18,144 patients with ACS to simvastatin 40 mg plus ezetimibe 10 mg daily or simvastatin alone [21] was the first trial to demonstrate improved CV outcomes using a statin plus a non-statin add-on. Simvastatin plus ezetimibe reduced the primary outcome of CV mortality, major CV event, or stroke by 34.7 vs. 32.7%; (*p* = 0.016) and demonstrated a reduction in MI and stroke. These results were likely due to LDL-C lowering, and this resulted in a change to guidelines to include ezetimibe therapy as an add-on to statin therapy. Furthermore, in 2018, a subgroup analysis of the 27% of patients in IMPROVE-IT with baseline diabetes suggested that the effect of ezetimibe was in fact most beneficial in patients with diabetes [22••], as demonstrated in Fig. 1. Indeed, if we take this figure at face value, it appears that the relative risk (RR) reduction from ezetimibe was evident only in those with T2DM. One suspects this is somewhat of a chance finding, but nevertheless, the striking findings in those with diabetes will lead to greater consideration of ezetimibe in diabetes when patients are either unable to tolerate a statin or when they are far from target values despite maximally tolerated statin dose.

#### PCSK9 Inhibitors

Proprotein convertase subtilisin/kexin type 9 (PCSK9) inhibitors are a modern class of monoclonal antibody injectable therapies developed to lower LDL-C by reducing the degradation of LDL receptors in the liver and increasing LDL-C clearance. Currently in the UK and other high-income countries, evolocumab and alirocumab are available for patients with familial hypercholesterolaemia with  persistently raised LDL-C, or very high-risk patients, and are given as injections either two- or four-weekly. Recent trial evidence clearly demonstrates their clinical efficacy. The FOURIER trial, a large randomized, double-blind, placebo-controlled trial conducted on high-risk patients demonstrated that evolocumab, in addition to statin therapy, lowered baseline LDL-C by 59% compared with placebo, with a corresponding reduction in CV risk (hazard ratio (HR), 0.85; 95% confidence interval [CI], 0.79 to 0.92; *p* < 0.001) [23]. The investigators have subsequently published their pre-specified analysis on the 16,533 patients with diabetes at baseline (40% of patients recruited in the FOURIER trial) demonstrating that evolocumab was beneficial in patients with diabetes and without, with similar levels of CV risk reduction; primary composite endpoint without diabetes HR 0.87 (95% CI 0.79–0.96; *p* = 0.0052) and with diabetes HR 0.83 (95% CI 0.75–0.93; *p* = 0.0008) (p_interaction_ = 0.60) [24•]. Pertinently, they also demonstrated that PCSK9 inhibition did not increase the risk of diabetes or worsen glycemic control. Similarly, the ODYSSEY trial looked at outcomes of alirocumab in addition to statin therapy in patients following an acute coronary syndrome (ACS). The authors reported occurrence of a primary end point event in 11.1% of placebo patients versus 9.5% of alirocumab-treated patients (HR 0.86 [95% CI 0.78–0.93], *p* < 0.001) [25]. They also conducted a pre-specified analysis on patients with diabetes, showing that the drug did not increase new-onset diabetes risk, and that a similar relative risk reduction was seen in diabetes patients; HR 0.84, (95% CI 0.74–0.97) [26].

To assess the efficacy on metabolic profiling of PCSK9 inhibitors compared with statins, a large, multi-centre collaboration utilized circulating metabolic measures in eight population cohorts (*N* = 72,185), using *PCSK9* rs11591147 as an unconfounded proxy to mimic the therapeutic effects of PCSK9 inhibitors. In this, we concluded that genetic inhibition of PCSK9 had similar metabolic effects to statin therapy on detailed lipid and metabolite profiles. However, PCSK9 inhibition was predicted to have weaker effects on the lowering of VLDL lipids compared with statins for an equivalent lowering of low-density lipoprotein cholesterol, potentially translating into slightly smaller reductions in CVD risk [27]. Of course, such a small difference is unlikely to be pertinent in very high-risk patients recommended for PCSK9 inhibitors in clinical practice, most of whom will have LDL-c levels well above targets.

### The Evidence Base for Fibrates in Type 2 Diabetes

Peroxisome proliferator-activated receptor-α (PPAR-α) agonists, or fibrates, are a class of drug used to lower TG levels and have a modest effect on raising HDL-C levels. They are generally considered add-on therapy to statins, but can be utilized alone, and although they are less effective at lowering total cholesterol, they can increase HDL-cholesterol and reduce TG levels more effectively than statins. The evidence base for use in diabetes is however limited in comparison to the strong and consistent statin evidence. The Helsinki Heart Study reported a significant reduction in CVD outcomes with gemfibrozil in men with dyslipidemia [28], but neither the Fenofibrate Intervention and Event Lowering in Diabetes (FIELD) study nor the ACCORD study showed a reduction in total CVD outcomes in studies looking specifically in patients with T2DM*.* The FIELD study did not demonstrate a difference in the primary composite endpoint of CHD death (*p* = 0.16), but it did show a reduction in nonfatal myocardial infarctions and revascularizations. It should be noted that a larger proportion of the placebo group was commenced on statin therapy, which may have attenuated the treatment benefit [29] in the fenofibrate group. Five years after FIELD, the ACCORD study group also failed to demonstrate a reduction in the rate of fatal CV events, nonfatal myocardial infarction, or nonfatal stroke when fenofibrate was added to simvastatin [30], with only a modest reduction of TG levels and an increase in HDL-C levels seen. Consequently, the use of these drugs is generally reserved as an add-on to statins for mixed hyperlipidemia.

There is continuing interest, however, in these drugs. Jun and colleagues suggested that although trial findings of the effects of fibrates were inconsistent, across the board, fibrates did demonstrate an approximately 10% RR reduction from major cardiovascular events and 13% RR reduction for coronary events. Therefore, this class of drug may play a role in high-risk individuals [31]; the PROMINENT study is currently investigating CV outcomes in patients with diabetes taking pemafibrate, a selective peroxisome proliferator activator modulator-α (SPPARM-α), which in pre-trial data was shown to reduce TG ~ 50%, increase HDL-C by 13–16% and increase LDL-C by up to 13% (0.4 g daily dose) [32]. The drug has a different structure from traditional PPAR-α agonists, but the rationale for the study remains: that lowering TG and inflammation will improve CV outcomes in high-risk patients [33]. Patients with T2DM (of longer than 12 weeks duration) with mild-to-moderate hypertriglyceridemia (TG 2.26–5.64 mmol/l) and low HDL-C levels (<=1.03 mmol/l), who are either receiving moderate-to-high-intensity statin therapy, have LDL-C ≤ 1.81 mmol/L or who are statin intolerant and have LDL-C ≥ 2.59 mmol/L, have been randomized to either pemafibrate therapy (0.2 mg twice daily) or placebo, with an intention to follow up over 3.75 years.

### Newer Agents Being Tested (See Also Table 1)

Over recent years, there has been a marked increase in effective drug therapies for dyslipidemia. Further to the progress that has already been made, new agents continue to be trialed for safety and efficacy. The REDUCE-IT trial published earlier this year demonstrated a primary endpoint event occurring in 17.2% of patients treated with 2 g of icosapent ethyl (an eicosapentaenoic acid (EPA) ethyl ester used to lower TG levels), compared with 22.0% of a placebo group (HR 0.75; 95% CI 0.68 to 0.83; *p* < 0.001). However, more patients were hospitalized for atrial fibrillation and had significant bleeding events in the treatment group than in the placebo group, although none were fatal [34]. Also, the actual effect on TG was extremely modest, such that many feel this drug is not working via lipid lowering per se but potentially via other mechanisms.

Similarly for LDL-C lowering, the CLEAR-Harmony trial published in March demonstrated significant LDL-C reduction with bempedoic acid, an ATP citrate lyase inhibitor, at 12 weeks. Treatment reduced the mean LDL cholesterol level − 16.5% from baseline (difference vs. placebo in change from baseline, − 18.1 percentage points; 95% CI, − 20.0 to − 16.1; *p* < 0.001). The safety profile was also favorable [35]. This reduction in cholesterol is modest, and it is likely that this agent will be combined with other drugs so that an era of dual or combined lipid-lowering drugs will soon be common place.

### What the Guidelines Recommend on When to Start Lipid Lowering and the Remaining Uncertainties

Following the 2004 CARDS study, such was the strength of evidence reducing CVD risk with the use of statins that there was an opinion that people with T2DM should be screened to be excluded from statin therapy. Most guidelines use a goal-centered approach, aiming to lower LDL-C as the primary target of dyslipidemia in T2DM. The recommended goal is usually to titrate LDL-C to < 1.8 mmol/L in secondary prevention, with consideration given to total CV risk. That noted, recent ESC diabetes and prediabetes, as well as dyslipidemia guidelines, have suggested even lower targets in those with diabetes and very high CVD risk to an LDL-C below 1.4 mmol/l [36, 37]. Total cholesterol should be titrated to < 4 mmol/L, and a TG level of < 1.5 mmol/L. In most countries, those with T2DM aged > 40 years are recommended for statins, regardless of baseline cholesterol. Intensive lipid-lowering therapy with atorvastatin 80 mg is recommended for patients with diabetes and CVD. Only in England and Wales have doctors been told to revert to risk scoring before statins are recommended in T2DM. The NICE guidelines suggest offering atorvastatin 20 mg for the primary prevention of CVD to people with T2DM who have a 10% or greater 10-year risk of developing CVD, assessed using QRISK2. Whether such risk scoring leads less people with diabetes to receive statins will be interesting to examine.

Recently, the USA moved away from a targeted approach, with the ACC/AHA guidelines published in Nov 2013 recommending that in patients with T2DM [38], therapy should either be high-intensity statin therapy if the patient with diabetes is considered high risk, or moderate-intensity therapy if low risk. This approach is broadly similar to ESC/EAS guidelines published in 2016 although they use targets to differentiate treatment intensities for patients with diabetes at differing risks for CVD. For example, those at high risk, LDL-C target < 2.6 mmol/L (< 100 mg/dL) or a reduction of at least 50% if the baseline is recommended, whereas for those considered at very high risk, an LDL-C target of < 1.8 mmol/L is suggested [38], although as noted above, targets have lowered further in the recent ESC lipid guidelines. In general, therefore, nearly all patients with T2DM should be on statins at some point in their lives. Notably, those who develop T2DM when younger [39•] have even greater excess CVD events and mortality and so absolute health gains from statins are likely greater. By contrast, those who develop diabetes in their 80s lose virtually no life years if compared to those without diabetes at the same age [39•].

### Type 1 Diabetes: Are We Undertreating with Statins?

It is curious to note that on average, people who develop T1DM lose around 12 years of life expectancy, whereas those who develop T2DM lose on average around 6 years. This might surprise many, given that people with T2DM are often more obese and have more lipid abnormalities than those with T1DM. However, the much earlier development of T1DM and the longer exposure to hyperglycemia per se is of paramount importance to future CV risk, and as such, people with T1DM experience on average 30 years more of hyperglycemia exposure. The pattern of disease risk is also worth examining, with potentially more atherosclerotic disease in T1DM driven by hyperglycemia and ensuing early renal damage versus what we now believe to be a combination of excess atherosclerosis and hemodynamically driven cardiorenal disease in T2DM, the latter driven more in part by the obesity and associated metabolic perturbances. Yet, clinical experience shows far less statin is prescribed to those with T1DM. We have also shown that those who develop T1DM when younger have the greatest excess risks for CVD [40]. Consequently, rather than waiting until a patient with T1DM develops evidence of renal disease before commencing statins, it may be prudent to consider statins for most with T1DM above the age of say 30 years rather than the 40-year age threshold in T2DM. This may be a controversial suggestion, but there is plentiful evidence statins are safe in such age groups and that they lower cholesterol well [41]. There is also less to fear from statin use in women than imagined since there is no clinical evidence of teratogenicity, although good practice would be cessation of statin in those women aiming for pregnancy.

### Statin Side Effects: Retry Statin Then Aim for Lower Doses or Alternative Statins

Statins are, sadly, much maligned in the media for causing considerable harm, in particular muscle aches. However, the trial experience is vastly different with a number needed to harm of 1 in 2000 to develop actual myositis [42]. Observational data show us that in fact well over two-thirds of people with apparent side effects are able to take the same dose of statin when repeated without side effects. If side effects do return, then lower dose of the same statin often works. There is also evidence for lower side effect rates with pravastatin or low dose rosuvastatin, and these should be considered for patients complaining of side effects on lower dose atorvastatin or simvastatin. Doses as low as rosuvastatin 2.5 mg twice a week can be given to lend confidence to patients worried about statin side effects. The dose can then be titrated up in step wise fashion in the vast majority who tolerate this regimen. The point being that there is no reason for the vast majority of patients, whether or not they have diabetes, who require statins not to be on them. A specific statement on statin intolerance in line with these comments was published in 2015 [43].

### Metabolic Control and Lipid Levels

In the clinical setting, there is some evidence that raised TG levels are associated with poor glycemic control. A large, Chinese-based study published last year showed that in 20,108 patients being treated with insulin for T2DM, elevated TGs were strongly associated with inadequate glycemic control; adjusted ORs (95% CIs) of having an HbA_1c_ ≥ 53 mmol/mol were 1.06 (0.98, 1.15), 1.35 (1.23, 1.48), and 3.12 (2.76, 3.53) for those with triglyceride levels in ranges of 1.70 to 2.29, 2.30, to 3.39, and ≥ 3.40 mmol/L, respectively, compared with those with triglyceride levels of < 1.70 mmol/L [44]. Thus, if TG levels are substantially elevated in patients with T2DM despite lipid-lowering therapy, improvements in glycaemic control, if abnormal, will help improve lipid levels.

## Conclusion

Diabetes is a major CVD risk factor, with plentiful evidence for the benefits of multifactorial risk targeting (see Table 2 for summary points). Statins lower both cholesterol and to a lesser extent TG levels, and they remain the cornerstone for CVD risk reduction in diabetes. The vast majority of patients with T2DM should be offered statin therapy at some point in their lives, except perhaps those who develop the disease when very old. At the very least, most should be on a moderate statin dose, with those at highest risk or with established disease given intensive statins and aiming for lower target LDL-C or Non-HDL-C levels. Ezetimibe has emerged as an excellent add-on option when targets are not reached with maximally tolerated statin dose or in the very small minority of cases when no statin is tolerated at any dose. For those at exceptionally high risk and continuing high LDL-c levels despite statins and ezetimibe, PCSK9 inhibitors are now available and help lower risk. The evidence base for triglyceride-lowering agents remains incomplete, but ongoing trials should help improve evidence base. If patients with diabetes on statins have continuing high triglyceride levels well above 5–10 mmol/l, then fibrates may help. However poor glycemic control should first be considered as a contributor and improved if possible. Finally, younger people with T2DM and more people with T1DM than is currently the case would benefit from statins given recent evidence showing far greater risk and loss of life years. Such patients have greatest excess risks for adverse CVD outcomes and experience greater life years lost. Therefore, they have the most to gain from such therapies. Future guidelines should consider this newer evidence.

**Table 2:** Summary points on lipid patterns and management in patients with diabetes

• Diabetes patients have a more atherogenic lipid profile which contributes to their excess risk for CVD—this is especially the case in younger onset type 2 diabetes who tend to be more obese at diagnosis and have higher triglyceride and lower HDL-c levels and therefore higher non-HDL-c. • Statins are first-line treatments in diabetes with the vast majority benefiting from statins, and the majority of countries recommend statins in diabetes without the need for a risk score. • As in the general population, if side effects occur with statins in patients with diabetes, they should be advised to retry the same statin at the same dose since most will be fine on retrial. If not, the dose can be lowered or alternative statin tried. Patients should be advised that the vast majority can take some form of statin without issues and trial and error will achieve the right statin for them. • Ezetimibe is an excellent add-on choice for patients with diabetes when they are not at target despite maximally tolerated statin dose or whether they are truly intolerant. • PCSK9 inhibitors work just as well in diabetes as in those without diabetes and with no evidence of glycemia dysregulation. They should be reserved for patients with diabetes are exceptionally high risk of CVD and elevated LDL-c levels despite maximally tolerated statin plus ezetimibe. • If patients with diabetes continue to demonstrate higher triglyceride levels despite statin therapy, then first consider secondary causes such as significant hyperglycemia or obesity. Fibrates can be considered in such patients to lower pancreatitis risk but notably, whether currently available fibrates lower CVD risk remains an open question. • Finally, as younger patients with both type 2 and type 1 diabetes lose the most years of life expectancy from their diabetes, more such patients should be offered statins earlier in the course of their lives. Future guidelines need to consider newer evidence with respect to age of diabetes onset and lifetime risks.	
